# Enterobius vermicularis in the male urinary tract: a case report

**DOI:** 10.1186/1752-1947-1-137

**Published:** 2007-11-14

**Authors:** Athanasios Zahariou, Maria Karamouti, Polyanthi Papaioannou

**Affiliations:** 1 "Elpis" Hospital, Urology Department, Volos, Greece; 2 "Elpis" Hospital, Microbiology Department, Volos, Greece

## Abstract

Enterobius vermicularis is an intestinal nematode of humans. Adults usually have low worm burdens and are asymptomatic. Ectopic infections in the pelvic area or urinary tract rarely occur in women. We report a case of the patient with mild voiding difficulties such as urgency, frequency, nocturia, dysuria, mild low back pain or perineal discomfort. The patient's prostatic secretions showed a large number of inflammatory cells and several eggs. The size and the shape of the eggs identified them as a group of E. vermicularis. On examination we found a soft palpable material which was 5 mm diameter in size and spherical shape. Palpation gave the impression of a tissue than a stone. An incision was performed and a 4 mm long living worm was found. The microscopic examination identified the worm as E- vermicularis. It is an extremely rare manifestation of enterobius vermicularis infection since an intestinal-breeding worm is rarely found in the male genital tract.

## Background

Enterobius vermicularis is an intestinal nematode of humans and its principal mode of transmission is direct contact between infected and uninfected persons. Human infections occur when the eggs in the infective stage are accidentally ingested in a contaminated environment. Although the majority of infections are asymptomatic, it induces bothersome symptoms in some cases. This condition is referred to as "enterobiasis" and it includes perianal itching and dermatitis [1].

Adults usually have low worm burdens and are asymptomatic. However, in children, particularly when there are heavy worm burdens, neurological symptoms such as nervousness, restlessness, irritability and distraction may occur, and these may influence on child growth [1]. Rarely ectopic infections in the pelvic area or urinary tract occur [2,3].

We report a case of the male adult patient with a prostatitis and a chronic pelvic pain syndrome due to Enterobius vermicularis which was successfully treated with antihelmintic regimens.

## Case presentation

A 65-year-old man presented to the Outpatient Department of Elpis Hospital, for evaluation of a chronic bacterial prostatitis. He reported mild irritative voiding difficulties such as urgency, frequency, nocturia, dysuria, a mild low back pain and perineal discomfort. Although there was no urethral discharge, the patient complained of local burning sensation on urination and an itching sensation in the urethra. The symptoms lasted for fifteen days.

A complete review of systems was unremarkable as well as his medical and family history. The patient's hemogram was normal without leucocytosis. The midstream urine showed microscopic hematuria, pyuria but no bacteriuria indentifying the organism infecting the urinary tract was present. The PSA values were elevated 7,6 ng/ml with a free PSA/total PSA ratio 39%. The transrectal ultrasonography revealed a cystic mass in the transitional zone of the prostate with a solid and a nodular inner portion.

**Figure 1:**
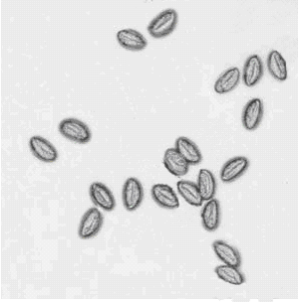
E. vermicularis eggs (wet mount).

**Figure 2:**
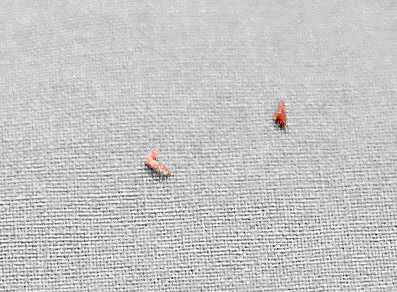
The living worm (left) after the incision of its spherical envelope (right).

On rectal examination, the prostate was normal in size and consistency. The prostatic secretions obtained by prostatic massage showed a number of inflammatory cells (more than 30 white cells per high-power field) but no causative infectious agent was found by culture or other means. Several eggs were observed amongst the inflammatory cells measured 51–59×26–29 μm with one convex side (Figure 1). The size and shape of the eggs identified them as a group of Enterobius vermicularis. On examination of the prostatic secretions we found a soft palpable material which was 5 mm diameter in size and spherical shape. Palpation gave the impression of a tissue than a stone. An incision was performed in this material and a 4 mm long living worm was found. (Figure 2). The microscopic examination identified the worm as Enterobius vermicularis.

Although there was a speculation that the infection was sexually transmitted, the patient denied it. The patient did not experience any symptoms from the gastrointestinal tract or itching sensation in the anus. The cello-tape test was performed and a microscopic examination identified Enterobius vermicularis eggs. Although the patient had a negative cello-tape test and no irritative symptoms he was treated with mebendazole 100 mg PO bid for 3 days followed by two more courses with 3 week time interval. At six month following evaluation of the patient, there has been no recurrence of enteroparasitosis.

After informing the patient, his three grandsons were examined using the cello-tape anal swab technique (one smear per child), for the presence of Enterobius vermicularis eggs. All three samples were positive for parasite eggs and the children were treated with the same antihelmintic regimen.

## Discussion

Enterobius vermicularis is one of the most frequently encountered and ubiquitous nematodes. It is highly contagious and parasitizing the human intestinal tract. The majority of human infections have been shown to occur in preschool children and grade school children, particularly those who engage in group activity.

It is known that the transmission of enteroparasites depends on the presence of infected individuals, sanitation deficiencies and, principally, the socioeconomic and cultural conditions of the population. Among adults, Enterobius vermicularis infection is similar in both sexes, but it was commonly observed that many adults seemed not to get infected even when subjected to contaminated environments due to personal hygiene [4].

According to our case, the urethra seemed the only route that the threadworm could have reached the urinary tract. Ordinarily, after a nocturnal egg laying excursion, the gravid female worms either die or return through the anus to their proper intestinal habitat. Occasionally, they lose their way and enter the vagina. They may then ascend to the genital tract, and even reach the peritoneal cavity along the lumen of the uterine tubes. Ovarian enterobiasis is rare but several cases have been recorded [5]. The uterine tubes and peritoneal serosa are the most common sites of ectopic enterobiasis. The majority of these cases have reported symptom of abdominal pain. Rarely, more serious disease can result, including weight loss, urinary tract infection and appendicitis [6]

Other orifices, whether it is natural or as result of a disease, may also mislead them. Surprisingly, there seems to be no indisputable record of threadworms entering the urethra, in spite of its accessibility. There are, however, a few published observations supporting the possibility that sometimes they may take this path, and these have been cited elsewhere [7,8]. Mainly, no good evidence has ever been produced that threadworms can penetrate through a healthy epithelial surface [9,10].

Effective antihelmintic regimens have been developed and used for decades. However, the control of enterobiasis is difficult due to frequent recurrences and a short life cycle. Therefore, continuous health education concerning improvement of personal hygiene and regular inspections are required to control enterobiasis.

## Conclusion

Repeated health education concerning improvement personal hygiene and regular inspections are required to control enterobiasis. Worm infection should be rarely suspected in patients with genitourinary symptoms or pelvic pain, in which a complete laboratory evaluation fails to establish the diagnosis.

## Competing interests

The author(s) declare that they have no competing interests.

## Authors' contributions

AZ was involved in the case directly and drafted part of the manuscript.

MK was involved in the literature review and helped draft part of the manuscript.

PP was involved in the case directly and performed the literature search and drafted part of the manuscript.
